# Drugs Targeting Epigenetic Modifications and Plausible Therapeutic Strategies Against Colorectal Cancer

**DOI:** 10.3389/fphar.2019.00588

**Published:** 2019-06-06

**Authors:** Srinivas Patnaik, Anupriya

**Affiliations:** School of Biotechnology, KIIT University, Bhubaneswar, India

**Keywords:** drugs, histone, colorectal cancer, therapy, epigenetics

## Abstract

Genetic variations along with epigenetic modifications of DNA are involved in colorectal cancer (CRC) development and progression. CRC is the fourth leading cause of cancer-related deaths worldwide. Initiation and progression of CRC is the cumulation of a variety of genetic and epigenetic changes in colonic epithelial cells. Colorectal carcinogenesis is associated with epigenetic aberrations including DNA methylation, histone modifications, chromatin remodeling, and non-coding RNAs. Recently, epigenetic modifications have been identified like association of hypermethylated gene Claudin11 (CLDN11) with metastasis and prognosis of poor survival of CRC. DNA methylation of genes CMTM3, SSTR2, MDF1, NDRG4 and TGFB2 are potential epigenetic biomarkers for the early detection of CRC. Tumor suppressor candidate 3 (TUSC3) mRNA expression is silenced by promoter methylation, which promotes epidermal growth factor receptor (EGFR) signaling and rescues the CRC cells from apoptosis and hence leading to poor survival rate. Previous scientific evidences strongly suggest epigenetic modifications that contribute to anticancer drug resistance. Recent research studies emphasize development of drugs targeting histone deacetylases (HDACs) and DNA methyltransferase inhibitors as an emerging anticancer strategy. This review covers potential epigenetic modification targeting chemotherapeutic drugs and probable implementation for the treatment of CRC, which offers a strong rationale to explore therapeutic strategies and provides a basis to develop potent antitumor drugs.

## Introduction

Colorectal cancer (CRC) is the third most common cancer in men and the second most common in women with expected increase in burden by 60% in the coming 10 years (Arnold et al., 2017). The mechanisms underlying CRC pathogenesis and progression remain subjects of extensive investigation in the field of cancer biology. It is known that CRC results from cumulation of both genetic and epigenetic alterations of the cellular genome drive transformation of normal glandular epithelium into adenocarcinoma. By further alteration in genetic and epigenetic profiles, CRC can acquire migration and invasion capability to metastasize to other parts of the body (Coyle et al., 2017; Hong, 2018). Epigenetic changes are defined as non-genetic influences on the gene expression. These changes do not include changes in DNA sequence but are inheritable. Epigenetic aberrations affect every aspect of tumor development from initiation to metastasis. Anomalous expression of genes including p53, Ras, beta-catenin, transcription factors involved in embryogenesis, and DNA mismatch repair genes drives the progression of the disease from benign adenoma to malignant adenocarcinoma (Vaiopoulos et al., 2014). Development of CRC is contributed by three major classical pathways of genomic instability—microsatellite instability (MSI), chromosomal instability (CIN), and CpG island methylator phenotype (CIMP) (Armaghany et al., 2012). CRCs that are positive for CIMP are rich in hypermethylation in CpG island of panel of marker genes CRABP1, SOCS1, RUNX3, MLH1, CACNA1G, NEUROG1, CDKN2A, and IGF2 (Bae et al., 2017). The aberrant methylation of tumor suppressor genes results in inactivation of these genes and subsequent promotion of neoplasia.

The field of epigenetics includes DNA modifications, histone modifications, and nucleosome remodeling. These modifications include methylation, acetylation, parylation, citrullination, phosphorylation, ribosylation, sumoylation, and ubiquitylation. Among these modifications, methylation and acetylation are widely studied. One of the main causes of morbidity due to cancer is the late detection. Methylation and acetylation status of few genes can be considered as potential epigenetic biomarkers for the early detection of CRC.

DNA methylation, one of the most important epigenetic phenomena, occurs in the cytosine of CpG dinucleotide islands and marks the inactivation of several key tumor suppressor genes required to drive the initiation and progression of CRC (Ichimura et al., 2015). Ezh2-mediated trimethylation of lysine 27 of histone 3 (H3K27me3) leads to inactivation of tumor suppressor genes and hence increases EMT phenotype and malignancy (Tiwari et al., 2013). Epigenetic modifications are also suggested as early-stage biomarkers for cancer. In the early stage of CRC, several tumor suppressor genes, CMTM3, SSTR2, and MDFI, are found to be remarkably hypermethylated in CRC tissues when compared with adjacent normal colorectal tissues (Li et al., 2017a). The DCC (Deleted in CRC) gene has repressive histone-tail marks, trimethylated lysine at the 9th and 27th position of H3 marked (H3K9me3 and H3K27me3) (Derks et al., 2009). Promoter regions of tumor suppressor genes hMLH1, MGMT, APC, and CDH1 were found to be hypermethylated in early stages of tumor formation in colon adenocarcinomas (Michailidi et al., 2015). The hypermethylated state of the MGMT (O^6^-alkylguanine DNA alkyltransferase) gene promoter, which is involved in DNA repair, is common in the case of brain metastasis from CRC and corresponding primary tumors (Maglio et al., 2015). Tumor suppressor gene MGMT, along with RASSF1A and FHIT gene promoter hypermethylation, is correlated with tumor stage and metastasis (Sinha et al., 2013). All of the abovementioned genes are involved in either tumor suppression or DNA repair, which, due to methylation, gets inactivated and hence results in uncontrolled and unchecked growth of cancer. Overall, methylation in certain genes is associated with more advanced tumor stage, poorly differentiated cancer cells, and metastasis.

Acetylation and deacetylation of histones and non-histone proteins are crucial events for gene regulation. Histone acetylation state is maintained by two crucial enzymes, histone acetyltransferases (HATs) and histone deacetylases (HDACs) (Verdone et al., 2006). Histone acetylation is an indicator of unconstrained expression of genes whereas deacetylation ensures repression in the gene expression. Therefore, HATs and HDACs are associated with hyperactivity and hypoactivity of genes, respectively. There are several evidences proving the significance of this kind of epigenetic modification in CRC. CNT2 (concentrative nucleotide transporter 2) is a pharmacologically important gene as it is a transporter that mediates the uptake of both natural nucleosides and nucleoside-derived drugs. However, CNT2 expression is significantly repressed in CRC due to hypoacetylation of the promoter region (Ye et al., 2018). One of the major regulatory cascades in the case of CRC is the Wnt/β-catenin signaling pathway, which is also controlled by epigenetic mechanism (Zhang et al., 2018). Cell-cycle-related and expression-elevated protein (CREPT), along with p300 (HAT), is involved in the acetylation of β-catenin, promoting oncogenic Wnt/β-catenin signaling and CRC (Zhang et al., 2018). There are many more cases reporting acetylation related to the progression of CRC. Scaffold Matrix Attachment Region Binding Protein 1 (SMAR1)-reduced expression is associated with poor prognosis of cancer. Loss of SMAR1 leads to enriched H3K9 acetylation of the β-catenin promoter that further activates the Wnt/β-catenin signaling pathway and CRC progression (Taye et al., 2018). One of the main reasons of the cancer relapse and drug resistance is cancer stem cells. Generation of cancer stem cells is associated with acetylation. The JADE3 (Jade family PHD finger 3) gene, which acetylates the histone during the transcription, was found upregulated in colon cancer cell line. Its overexpression increases while knockdown decreases the stem-cell-like property of colon cancer cells both *in vivo* and *in vitro*. Reduction in expression of JADE3 impairs the tumor-initiating property of cancer cells *in vivo*. JADE3 interacts with the promoters of LGR5 (colon stem cell marker) and activates its transcription by increasing the occupancy of p300 acetyltransferase and histone acetylation, hence substantially inducing Wnt/β-catenin signaling (Jian et al., 2018). Overexpression of HDACs has been shown to promote migration, invasion, tumorigenesis, and metastasis. HDAC has also been suggested to modulate the expression of IL-10 as a transcriptional activator (Cheng et al., 2014). Due to the fundamental significance of HDAC for cellular de-differentiation processes, HDAC inhibition has been proposed as a strategy to re-balance transcription of those genes deregulated in cancer.

In recent years, our understanding has been improved regarding the role of epigenetic factors in cancer. The inherent plastic nature of epigenetic changes provides possible lines for targeted treatment using specific inhibitors for proteins involved in epigenetic modifications. FDA has approved several of these compounds for the treatment of certain cancers, for example, azacytidine (market name Vidaza), which is a nucleoside-like compound. This drug acts as a cytidine analog; carbon at the fifth position is replaced by a nitrogen atom. During replication, azacytidine gets incorporated into DNA. It is recognized by DNMT1 and forms an irreversible DNMT1–aza linkage that triggers DNMT1 degradation and leads to overall reduction in methylation (Garcia-Manero et al., 2019; Nehme et al., 2019). HAT and HDAC inhibitors have also been identified to inhibit the catalytic activity of both in many cancer types. P300 is one of the potential histone acetylases that acetylates β-catenin in many cases of CRC (Zhang et al., 2018). A number of synthetic as well as natural HAT inhibitors (HATi) are used to inhibit p300, including bisubstrate inhibitors, garcinol, anacardic acid, C646, and natural HATi curcumin. HATi are less selective and bind many non-specific targets, whereas HDAC inhibitors (HDACi) are relatively more specific in nature. There are so many potential HDACi in the market. Hydroxamic acid has emerged as a potent chemotherapeutic drug that inhibits Class I and II HDACs. In fact, suberanilohydroxamic acid (SAHA) has been shown to inhibit HDAC at very minimal concentration (Guo and Zhang, 2012). This review covers potential epigenetic targeting chemotherapeutic drugs or the compounds that have the potential to be used as chemotherapeutic drugs for the restriction and treatment of CRC.

## Regulation of Colorectal Cancer by Epigenetic Mechanism

CRC is the third most common cancer in men and the second most common cancer in women worldwide. For decades, gene mutations have been known to be a significant contributor for cancer development. However, epigenetic changes have recently been shown to play a potential role in cancer progression, specifically in the case of CRC where it is demonstrated that epigenetic modifications act earlier than the genetic modifications in the progression of CRC (Porcellini et al., 2018). Development of CRC is contributed by three major classical pathways of genomic instability—MSI, CIN, and CIMP (Armaghany et al., 2012). CRCs that are positive for CIMP are rich in hypermethylation in CpG island of panel of marker genes CRABP1, SOCS1, RUNX3, MLH1, CACNA1G, NEUROG1, CDKN2A, and IGF2 (Weisenberger et al., 2006). Compendious genomic and epigenomic studies have revealed the heterogeneity of CRC. Epigenetic status of eight CIMP marker genes also correlates with heterogeneity of CRC. CRCs with five or six methylated genes compared with CRCs with less than five methylated genes showed moderate increase in MLH1 methylation, an MSI-high status, CK7 overexpression, and downregulation in expression of CK20 and CDX2. CRC cases with seven or eight methylated CIMP marker genes that showed overexpression of CK7 and downregulation of CK20 and CDX2 showed high incidences of *BRAF* mutations and lacked *KRAS* mutations. Based on these trends, CRCs were classified into CIMP-negative (CIMP-N, 0–4 methylated genes), CIMP-positive 1 (CIMP-P1, 5–6 methylated genes), and CIMP-positive 2 (CIMP-P2, 7–8 methylated genes) categories (Bae et al., 2017). Wnt/β-catenin signaling has been involved in a variety of cancers and other diseases. Loss of the Wnt signaling negative regulator adenomatous polyposis coli (APC) is the major hallmark of human CRCs (Novellasdemunt et al., 2015). APC is a tumor suppressor that blocks transition of cells from G_1_ to S phase. Stem cells reside at the base of the colonic crypts, which is maintained in their native undifferentiated state through Wnt/β-catenin signaling. These stem cells are responsible for the survival of normal stem cells as well as cancer stem cells. β-Catenin regulates the migration of stem cells out of epithelial crypts as they get differentiated. During this process, many stem cells acquire mutations. But in healthy people, these cells normally get sloughed off in a week after apoptosis and hence don’t get enough time to induce cancer. APC gene downregulates the Wnt/β-catenin signaling through its ability to bind β-catenin and mediate its degradation. Hypermethylation of the promoter region of APC causes its inactivation, which leads to the accumulation of β-catenin. Accumulation of β-catenin in enterocyte precursor results in retention of a stem cell phenotype, which prevents them from migrating to the surface to be sloughed off. The accumulation of these undifferentiated cells eventually leads to polyp formation in the colonic crypts (Armaghany et al., 2012). Mismatch repair gene promoter hypermethylation has been frequently observed in sporadic CRC with MSI while hypermethylation of the APC promoter is positively correlated with CRC metastasis (Van Engeland et al., 2011; Roy and Majumdar, 2012). Another DNA-repair gene, MGMT, is found silenced in CRC due to hypermethylation of the promoter region, which favors mutation in p53 and kRAS genes. MGMT promoter hypermethylation state can be seen at precancerous polyps (Matsubara, 2012). This finding suggests that hypermethylation of certain genes occurring at the early stage of CRC could provide promising diagnostic biomarkers. Hypermethylation-mediated silencing of genes in CRC has been widely studied. However, recently, there have been many reports mentioning the involvement of histone modification in CRC progression, in particular acetylation of lysine residues of H3 and H4. Acetylation level is regulated and balanced by the function of both HAT and HDAC activity (Dawson and Kouzarides, 2012). Specifically, the HDAC family of proteins is commonly upregulated in CRC, including HDAC1, HDAC2, HDAC3, HDAC5, and HDAC7 (Barneda-Zahonero and Parra, 2012). HDAC2 upregulation was observed as one of the earliest events in CRC carcinogenesis, which may serve as an early-stage biomarker for detection (Stypula-Cyrus et al., 2013). HDACs are responsible for silencing of tumor suppressor genes. Wnt/β-catenin target genes CDX1 and EPHB act as tumor suppressors in intestinal epithelial cells, which are frequently found to be downregulated in CRC. Study showed involvement of HDAC1 and HDAC3 that caused strong reduction of active histone modifications in the promoter region of CDX1. Unlike the inactive CDX1 locus, EPHB encoding DNA was hypomethylated in the promoter regions in silent state. Treatment with both DNMT and HDACi restored the tumor suppressor genes’ activity (Rönsch et al., 2011).

### Histone Deacetylases Inhibitors

**Figure 1:**
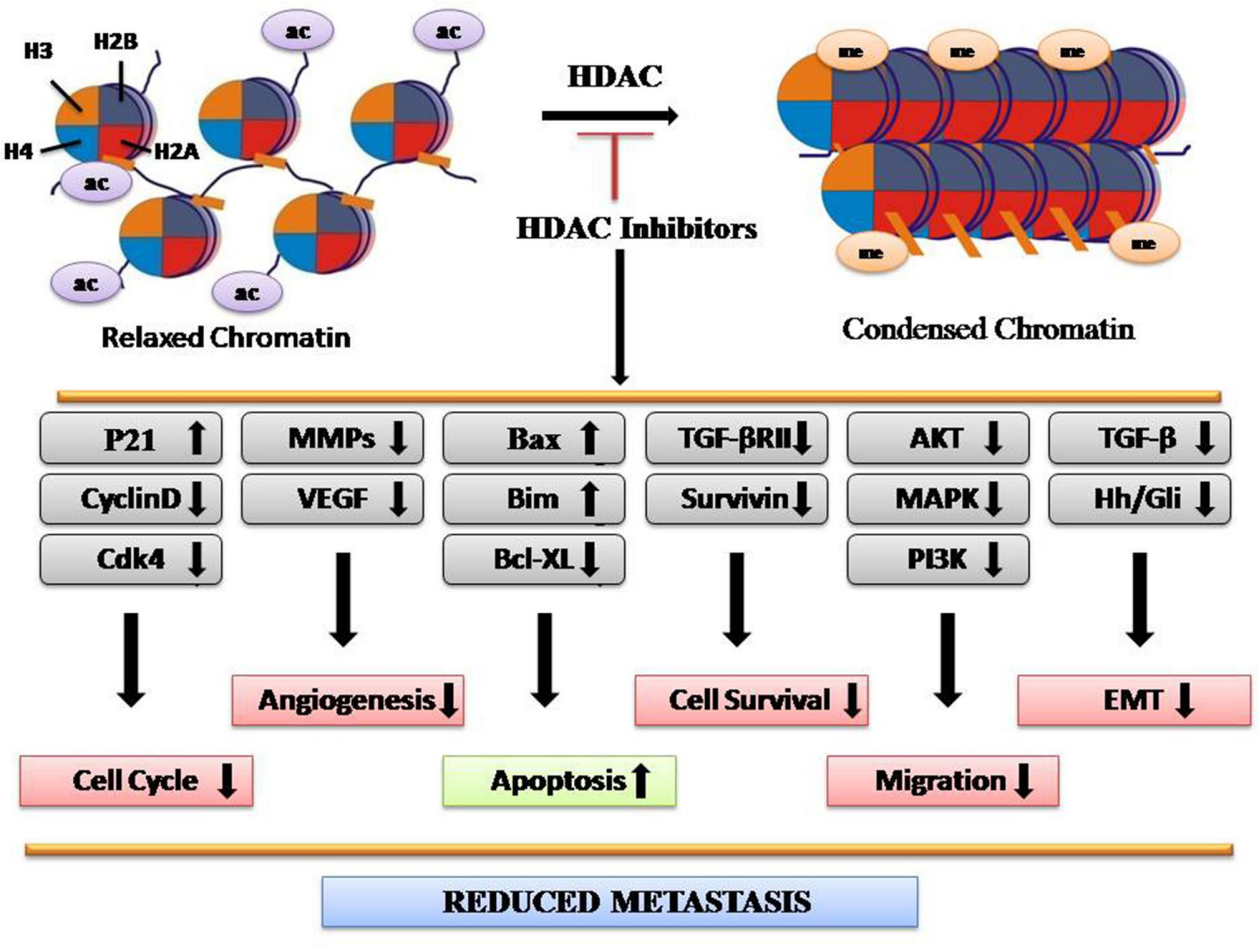
Depiction of molecular mechanism of HDAC inhibitors as anticancer agents.

HATs mediate acetylation of amino acid residues within histone tails, which lose chromatin structure, thus making target genes more accessible for transcription factors. Conversely, HDACs catalyze histone deacetylation, resulting in chromatin condensation and transcriptional repression (Eberharter and Becker, 2002). The HDAC family consists of 18 members, subdivided into four classes: I (HDAC1–HDAC3 and HDAC 8), II (HDAC4–HDAC7, HDAC9, and HDAC10), III (sirtuins 1–7), and IV (HDAC11) (Barneda-Zahonero and Parra, 2012). Upregulated expression of HDAC2 is found in the early stages in CRC along with HDAC1–3, HDAC5, and HDAC7 (Stypula-Cyrus et al., 2013). Expression levels of HDACs vary according to the tissue type. Upregulation of HDAC1 has been seen in prostate, gastric, lung, esophageal, breast, and colon cancer. HDAC2 was found upregulated in cervical, gastric, and CRC, whereas HDAC6 and HDAC11 expressions are found more in breast cancer and rhabdomyosarcoma, respectively (Eckschlager et al., 2017). HDAC-regulated proteins include STAT3, tumor protein p53, Myc, RUNX3, β-catenin, EKLF, estrogen receptor, GATA family, HIF-1α, Foxp3, NF-Κb, and MyoD, which play major roles in cancer progression (Hull et al., 2016). Studies have shown that HDAC1 inhibition controls the proliferation and inhibits tumorigenecity, while HDAC3 inhibition reduces cell migration with the overexpression of epithelial markers (Hayashi et al., 2010). Several lines of evidences have demonstrated that the aberrant expression of HDACs is often associated with poor prognosis as well as poor survival rates in CRC. HDACi have emerged as novel drugs with potent anticancer activity in both preclinical experiments and clinical trials. For example, Vorinostat (SAHA), romodepsin, belinostat, and panobinostat are the potent HDACi, approved for cancer therapy by FDA (Eckschlager et al., 2017). Overall, HDACi reduce metastasis by reducing the expression of genes involved in migration, angiogenesis, epithelial-to-mesenchymal transition (EMT), and cell survival while enhancing the expression of genes involved in apoptosis (**Figure 1**).

HDACi may be specific or act against all types of HDACs. HDACi can be classified into five categories on the basis of nature of compounds—hydroxamic acids, short chain fatty acids, benzamides, cyclic tetrapeptides, and sirtuin inhibitors (Ceccacci and Minucci, 2016). Currently, there are a number of HDACi that are in use or under clinical trials.

### Sulforaphane

Sulforaphane (SFN) is a natural compound found in cruciferous vegetables from the Brassicaceae family like broccoli, cauliflower, Brussels sprouts, and cabbage. It comes within the isothiocyanate group of organosulfur compounds. Various clinical and epidemiological studies have proven SFN as a potential chemopreventive agent, which can be used for cancer treatment (Michaud et al., 1999; Cipolla et al., 2015). It has shown prodigious antitumor effects *in vitro* and *in vivo* without any toxic effect (Alumkal et al., 2015). SFN is well known to suppress HDACs, which alter epigenetic regulation. The interaction of SFN and HDAC has been shown both clinically and preclinically. The formulation of this drug was based on the previous findings about the anticancer properties of its sources like broccoli. In the past, there is a study on human subjects showing significant inhibition of HDAC activity following consumption of a single dose of 68 g of broccoli sprouts (Dashwood and Ho, 2007). SFN effect has been proven *in vivo* too. In a prostate cancer xenograft mice model, the daily consumption of 7.5 µmol SFN per mice for 21 days significantly decreased HDAC activity, indicated by an increase of acetylated histones. These findings suggested SFN activity in suppressing the cancer cell growth through increased histone acetylation (Myzak et al., 2007). Feeding colon cancer xenograft mice models with 10 µmol SFN per mice gave the same result. It was concluded that inhibition of HDAC activity coupled with increased acetylated histones might contribute to the cancer chemoprotective and therapeutic effects of SFN (Myzak et al., 2007). In a few SFN-treated prostate cancer cell lines, several class I and class II HDAC (HDAC3, HDAC4, HDAC6, and HDAC8) protein levels decreased, whereas in normal cells, only a temporary depletion of HDAC activity was noticed. It revealed the selective function of SFNs for benign hyperplastic and cancer but not normal cells (Clarke et al., 2011). SFN convincingly targets HDAC1, HDAC2, HDAC3, and HDAC8, but not HDAC6 in CRC cells (Juengel et al., 2018).

Focusing on SFN molecular mechanism, it has all-around chemopreventive properties. SFN by its HDAC inhibiting property alters apoptotic and cell cycle regulating gene expression, which blocks growth of tumor cells and induces apoptosis *in vitro* as well as *in vivo*. SFN triggers checkpoint kinase 2 (Chk2) phosphorylation-dependent upregulation of p21, which inhibits cyclin-dependent kinase (cdk), and results in cell cycle arrest. In prostate cancer cells, cyclin D1, cyclin E, Cdk4, and Cdk6 protein level reduction correlated with S phase arrest induced by SFN treatment, whereas activation of the G(2)-M checkpoint correlated with induction of cyclin B1 and reduction of Cdk1 and mitosis inducer Cdc25C protein levels (Herman-Antosiewicz et al., 2007). Advanced stage cancers are characterized by metastasis, which includes cell migration, invasion, and angiogenesis. It is therefore interesting that SFN intervenes with the cancer cells’ invasion cascade and angiogenesis by downregulating matrix metalloproteinases (MMP) such as MMP-1, MMP-2, MMP-7, and MMP-9 (Shankar et al., 2008). In recent years, the role of microRNAs (miRNAs) has been acknowledged in the regulation of gene expression at the epigenetic level, hence causing cell development and progression of various cancers. SFN has been shown to regulate certain miRNAs like miRNA-21 and miRNA-320a (Sato et al., 2016; Martin et al., 2018). Although a clear connection between SFN and miRNAs has not been deciphered yet, it gives further scope for investigation as these miRNAs are involved in the progression of EMT, which leads to aggressiveness of tumors (Yang et al., 2018).

### Vorinostat

Vorinostat, also known as suberoylanilide hydroxamic acid (SAHA), is an orally bioavailable broad HDAC inhibitor and commonly inhibits HDAC class I and II. It is a new potential therapeutic drug used for the treatment of cancer (Richon et al., 1998; Bubna, 2015). Vorinostat does not inhibit HDAC class III enzymes (Richon et al., 2000). United States Food and Drug Administration (FDA) approved it as the first HDACi that is used for the treatment of relapsed cutaneous T-cell lymphoma (CTCL) (Eckschlager et al., 2017). SAHA plays a role both *in vitro* and *in vivo*, induces apoptosis of cancer cells *in vitro*, as well as inhibits tumor growth in mouse models (Glick et al., 1999; Richon et al., 2000; Suraweera et al., 2018). It has also been used in combination with other drugs like tamoxifen, pembrolizumab, sorafenib, rituximab, and gefitinib for cancer treatment (Richon et al., 2000).

Previous studies have shown the SAHA involvement in cell cycle arrest. The expression of the cyclin-dependent kinase inhibitor WAF1 induced by SAHA leads to T24 bladder carcinoma cells’ growth arrest (Butler et al., 2002). SAHA increases expression of TATA Box binding protein-2 (TBP-2) that inhibits thioredoxin, which is an intracellular antioxidant in prostate, bladder, and breast cancer cells. Treatment of cancer cells by this HDAC inhibitor induces ROS-dependent apoptosis (Lincoln et al., 2003; Lin and Pollard, 2007). Vorinostat acts indirectly under hypoxic conditions, suppressing hypoxia inducible factor (HIF)-1 alpha and vascular endothelial growth factor (VEGF), and thus blocks angiogenesis (Zhijun et al., 2016). HDAC overactivity is known in CRC progression. Hence, SAHA can be used to target HDAC for CRC treatment.

### Domatinostat (4SC-202)

4SC-202 (domatinostat) is an orally administered small molecule for the treatment of various types of cancer. The compound inhibits the enzymes HDACs HDAC1, HDAC2, and HDAC3, which are believed to play important roles in the regulation of aberrant cancer signaling. It potently inhibits survival, proliferation, and cell cycle progression in CRC cells (HT-29, HCT-116, HT-15, and DLD-1). The colon epithelial cells that have low expression of HDAC1/2 had minimal effect on them after 4SC-202 treatment. This result showed the specificity of 4SC-202 for HDACs (Maes et al., 2015). It has dual HDAC and KDM (lysine demethylases) inhibitory activity (Gruber et al., 2018). Together, these preclinical results indicate that 4SC-202 may be further investigated as a valuable anti-CRC agent/chemo-adjuvant.

Domatinostat targets the oncogenic Hedgehog (HH)/Gli signaling pathway and hence reduces proliferation, survival, self-renewal, metastasis, and overall tumor formation and cancer progression (Fu et al., 2016). Addition of 4SC-202 in hepatocellular carcinoma (HCC) cells activates ASK-1 dependent mitochondrial apoptosis pathway (Mishra et al., 2017). 4SC-202 treatment inhibits TGFβ-induced EMT. It markedly induces p21 expression and significantly attenuates cell proliferation. Genome-wide studies revealed that 4SC-202-induced genes were enriched for Bromodomain-containing Protein-4 (BRD4) and MYC occupancy (Zhao et al., 2018).

### Resminostat (4SC-201)

Resminostat (4SC-201 or RAS2410) is an orally bioavailable inhibitor of HDACs. It is a direct inhibitor for HDAC classes I and II including HDACs 1, 3, 6, and 8. It was shown to reduce the growth of HCC cells by inhibiting the proliferation with its specificity for class I HDACs (Mandl-Weber et al., 2010). Resminostat was seen to have anti-myeloma activity (Brunetto et al., 2013). The effect of Resminostat has been seen on several cancers including head and neck squamous cell carcinoma (HNSCC), multiple myeloma (MM), and HCC. In human patients with advanced solid tumors, an investigation on Resminostat was carried out to study its safety and tolerability. It was found to be safely administered and to have anticancer effects with a dose-proportional pharmacokinetic profile (Tambo et al., 2017). Resminostat is also used with the combination of other drugs for better efficacy like sorafenib and docetaxel (Bitzer et al., 2016; Mandl-Weber et al., 2010).

Resminostat inhibits proliferation and induced G_0_/G_1_ cell cycle arrest along with decreased levels of cyclin D1, cdc25a, Cdk4, and pRb, as well as upregulation of p21. It strongly induces apoptosis in MM cell lines shown by increased expression level of Bim and Bax and decreased expression level of Bcl-xL (Enzenhofer et al., 2017). In the case of MM and HNSCC, Resminostat reported to have anticancer activity by affecting the AKT signaling pathway and hence reducing cell survival and proliferation (Soukupova et al., 2017). Resminostat prevents cell growth and induces death in HCC cell lines with a decrease in the mesenchymal related genes and an increase in epithelial-related genes. Moreover, it downregulates the CD44 expression and hence the colony formation capacity of HCC cells (Gimsing et al., 2008).

Resminostat is in clinical trials for the treatment of advanced CRC but no results have been published yet. Activation of AKT signaling in the CRC results in proliferation, migration, and inhibition of apoptosis in CRC cell lines, which suggests the possible usage of Resminostat for CRC treatment.

### Belinostat

Belinostat is a hydroxamate acid-type HDAC inhibitor (HDACi) drug with antineoplastic activity. It was developed by TopoTarget for the treatment of hematological malignancies and solid tumors. Importantly, it has tolerable side effects with infrequent toxicity when compared to other HDACi (Beck et al., 2010). Human colon cancer cell line HCT116 proteomic profiling has been done to evaluate the effect of this drug on the expression of proteins involved in cancer progression and regression. In Belinostat-treated cells, 45 differentially expressed proteins related to proto-oncogene were revealed, including nucleophosmin and stratifin, which were downregulated, and nucleolin, gelsolin, heterogeneous nuclear ribonucleoprotein K, annexin 1, and HSP90B, which were upregulated (Tumber et al., 2007). Belinostat in combination with the other chemotherapeutic drug 5-fluorouracil has shown promising effect to inhibit colon cancer cell growth *in vitro* and *in vivo* (Kong et al., 2017). Belinostat has promising chemosensitizing characteristics in lung squamous cell carcinoma. Its treatment triggers the proteasomal degradation of SOS proteins FBXO3 and FBXW10 through the suppression of MAPK activity (ERK1/2 and p38) (Chowdhury et al., 2011). This drug has been tested on colon, breast, and pancreatic cancer cells with an epigenetically silenced TGFβ receptor. Belinostat induces the expression of tumor suppressor gene TGFβRII with simultaneous restoration of the downstream cascade. Survivin, a cancer-associated gene, gets downregulated through TGFβ/protein kinase A (PKA) pathway. This results in poor cell survival and reduced metastasis. Hence, its downregulation leads to cancer cell death (Giles et al., 2006).

The drug is still being investigated for its effective use against multiple cancers. Preclinical experiments demonstrated that the drug works by inhibiting cell proliferation and inducing programmed cell death in tumor cells.

### Panobinostat

Panobinostat (LBH589) is a member of the hydroxamic acid class of HDACi approved by the FDA for the treatment of MM. It is a colorless, clear, slightly viscous liquid and chemically known as (2E)-*N*-hydroxy-3-[4-[[(2-hydroxyethyl) [2-(1H-indol-3-yl)ethyl]amino]methyl]phenyl]-2-propanamide, administered in both oral and intravenous forms (Atadja, 2009). It is a nonselective HDAC inhibitor that works against all the classes of HDACs including class I, II, and IV. It interferes with both histone and non-histone proteins. Panobinostat treatment increases acetylated H3 and H4 as well as non-histone proteins HIF-1α, α-tubulin, β-catenin, chaperons (HSP90), estrogen receptor (ERα), androgen receptor (AR), signaling mediators (Stat3, Smad7), DNA repair proteins (Ku70), retinoblastoma protein (pRb), etc., leading to alterations in transcriptional factors (p53, E2F, NF-кB, c-Myc) (Singh et al., 2010; Kim and Bae, 2011; Jones et al., 2011).

It has been tested for several types of cancer including hematologic and solid malignancies, CTCL, Hodgkin’s lymphoma, leukemia, prostate, thyroid, and breast cancer (LaBonte et al., 2009). One of the main advantages of Panobinostat is its ability to prolong hyperacetylation of histones, which allows intermittent dosing schedules to reduce the challenging thrombocytopenia. Thrombocytopenia is a condition that defines low platelet counts, which is more likely a side effect of all HDACi. Hence, Panobinostat is currently a more potent HDAC inhibitor with better persistence in the body. Panobinostat treatment of colon cancer cell lines inhibits proliferation and survival at nanomolar concentrations. Analysis of gene expression profiles of CRC cell lines treated with panobinostat revealed alteration of genes involved in the process of angiogenesis, mitosis, DNA replication, and apoptosis (Regel et al., 2012).

Panobinostat, when used in combination with anthracyclines, works as a chemosensitizing agent for gastric cancer cells *via* activation of CITED2 (Cbp/p300-interacting transactivator 2) (Catalano et al., 2012). Panobinostat induces G1, G2/M cell cycle arrest and cell death in the case of head and neck cancer and CRC respectively (Prystowsky et al., 2009; Gandesiri et al., 2012). Combined treatment of lapatinib and panobinostat inhibits the proliferation and colony formation in all CRC cell lines tested. Lapatinib is an EGFR/HER2 kinase inhibitor. Combination treatment resulted in rapid induction of apoptosis with increased DNA double-strand breaks, caspase-8 activation, and PARP cleavage with downregulation of transcriptional targets including NF-κB1, IRAK1, and CCND1. This was paralleled by decreased signaling through the MAPK and PI3K/AKT pathways.

In CRC, panobinostat has been shown to activate the tumor suppressor gene death-associated protein kinase (DAPK), which plays a role in induction of autophagy and apoptosis (LaBonte et al., 2011). Panobinostat treatment downregulates EGFR, HER2, and HER3 expression both at the mRNA and protein level through transcriptional and posttranslational mechanisms (Costello and Plass, 2001).

### DNA/Histone Methyltransferase Inhibitors

**Figure 2:**
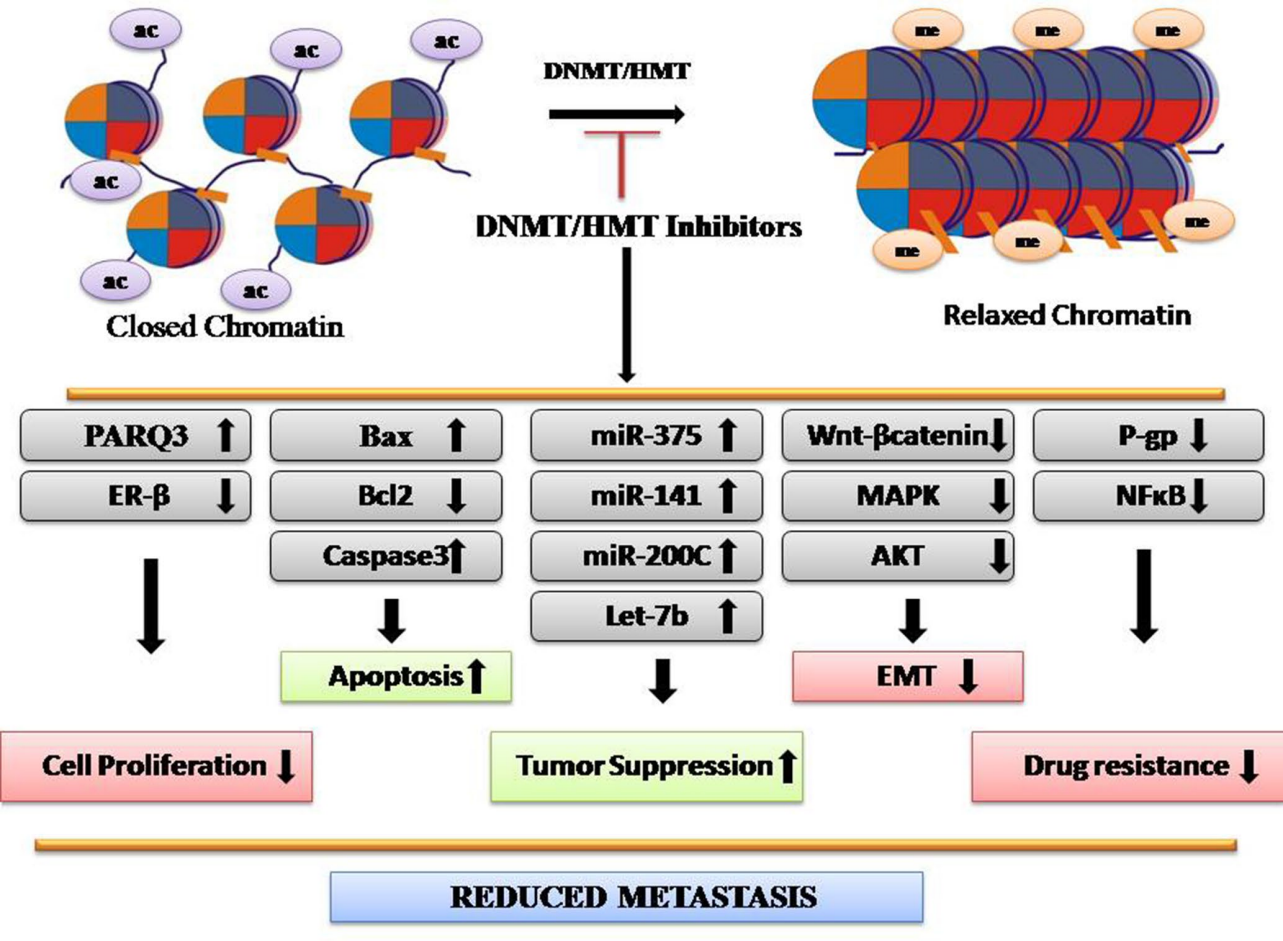
Depiction of mechanism of DNMT/HMT inhibitors as anticancer agents.

Methylation is involved in several biological processes, including developmental, cell cycle, differentiation, and DNA repair (**Figure 2**). Any alteration in methylation can affect any of these events and results in disease development. H3K4me2 is downregulated in many types of cancers including lung, kidney, prostate, breast, and pancreatic cancer, whereas H3K27me3 is downregulated in gastric adenocarcinoma and downregulation of H4K20me3 is associated with CRC (Greer and Shi, 2012). Epigenetic silencing by aberrant methylation of regulatory genes leads to tumorigenesis. Inactivation of critical genes involved in tumor suppression, DNA repair, cell-cycle regulatory mechanism, apoptosis, angiogenesis, and metastasis has been demonstrated in a wide variety of tumor types (Orta et al., 2017). Enzymes responsible for these processes are histone methyltransferases (HMTs) and DNA methyltransferase (DNMT) that maintains an altered methylation pattern by copying it from parent to daughter DNA strands after replication. DNMT1 high expression is observed in almost all cancer types and maintains a higher methylation level (Heerboth et al., 2014). Aberrant epigenetic changes induced in malignant cells lead to emergence of neoplastic properties. Deviant histone methylations have been suggested to play a major role in CRC. More than 20 histone methylation enzymes are found to be clinically relevant to CRC, including 17 oncoproteins and 8 tumor suppressors (Huang et al., 2017). However, abnormal epigenetic patterns can be reversed by the action of epigenetically active agents. In the recent years, CRC epigenetic regulation, particularly HMTs and demethylases (HDMs), has been the subject of extensive research. Agents used as HMT and DNMT inhibitions have shown promising anticancer effects. For example, EZH2 and DOT1L inhibitors have shown potency in preclinical trials for CRC treatment. Chaetocin, a fungal metabolite, inhibits SUV39H1 and inhibits migration of CRC cells (Yokoyama et al., 2013). Altogether, DNMT/HMT inhibitors reduce metastasis (**Figure 2**).

Aberrant histone and DNA hypermethylation is frequently found in tumor cells, and inhibition of methylation is an effective anticancer strategy. Currently, there are a number of DNMT/HMT inhibitors used as chemotherapeutic drugs for CRC treatment.

### Zebularine

Zebularine [1-beta-D-ribofuranosyl-2(1H)-pyrimidinone] is a hydrophilic, orally bioavailable nucleoside analog of cytidine. It inhibits DNA methylation by getting incorporated into DNA, hence appealing for use in rapidly dividing cancer cells. Zebularine acts as an inhibitor of DNA methylation by inhibiting the action of DNMTs. Zebularine-substrate DNA forms covalent bond with DNMTs and gets entrapped in the complex (Zhou et al., 2002). It possesses high oral bioavailability and shows low toxicity and high efficacy, being a promising adjuvant agent for anticancer chemotherapy (Cao et al., 2018). In addition, the low toxic effect of zebularine gives scope for low-dose administration for a prolonged period.

Zebularine-treated cumulus cells were found with reduced overall DNA methylation patterns and gene-specific DNA methylation levels at the promoter regions of pluripotency genes (Oct4, Sox2, and Nanog), which indicates that zebularine has a role to play in the case of cancer stem cells (Rao et al., 2007).

Several studies have reported its antitumor effects on several types of cancers like lung cancer, gastric cancer, pancreatic cancer, medulloblastoma, leukemia, head and neck cancer, hepatocellular carcinoma, cervical cancer, breast cancer, bladder carcinoma, prostate cancer, ovarian cancer, and CRC. Zebularine was developed to counter the shortcomings of DNMTI 5-azacytidine. Zebularine was reported to be safer than 5-azacytidine for the treatment of cancers in Epstein–Barr Virus (EBV) carriers and proposed to be used against tumors possessing EBV (Takemura et al., 2018).

In human malignant mesothelioma cells, DNMT1 expression decrease was directly proportional to zebularine. It exerted antiproliferative activity through S phase delay and cell death (You and Park, 2014). It has been seen to have the same effect in the case of lung cancer and induces A549 cell death, which was accompanied by the loss of mitochondrial membrane potential, Bcl-2 reduction, and activation of Bax, p53, caspase-3, and caspase-8 (Nakamura et al., 2015). It induces suppression of the Wnt signaling pathway by decreasing β-catenin protein levels in cholangiocarcinoma (CCA) cell lines TFK-1 and HuCCT1, which leads to apoptotic cell death in CCA (Yang et al., 2013). These studies indicated that zebularine could effectively target both DNMT inhibitors and non-DNMT inhibitors. In lymphoma cells, zebularine reactivates silenced E-cadherin, which suggests its capacity to reverse the EMT (Takemura et al., 2018).

The effect of zebularine on CRC has also been investigated. It induces p53-dependent ER stress and autophagy, whereas it inhibits tumorigenesis and stemness of CRC (You and Park, 2014). Zebularine also works at the mRNA level. Its treatment increases the expression level of let-7b, which functions as tumor suppressor microRNA and hence suppressed the invasion activity of CRC cells (Tanaka et al., 2017).

Genes involved in tumor suppression, DNA damage repair, and cell cycle regulation often get inactivated because of CpG island hypermethylation, which causes cancer progression. Methylation inhibitors target the transcriptional silencing of tumor suppressor genes due to hypermethylation and direct the reactivation. Zebularine is one such methylation inhibitor with the potential for clinical utilization with less cytotoxic effect, stability, and high selectivity for cancer cells, making it a promising candidate as a chemotherapeutic agent (Yoo et al., 2004; Veverka et al., 1997).

### Disulfiram

DSF (DSF, bis-diethylthiocarbamoyl disulfide) also known as Antabuse, is an irreversible inhibitor of aldehyde dehydrogenase (ALDH), which is responsible for ethanol metabolism. It contains a thiol-reactive functional group that interacts with a thiol group at the active site of ALDH and hence it is used for the management of alcoholism (Lin et al., 2011). DNA methyltransferase 1 (DNMT1) contains a reactive CXXC region (C is cysteine; X is any other amino acid) at its active site, which makes it susceptible to DSF (Syro et al., 2006). Disulfiram has the ability to cross the blood–brain barrier and has been reported as a potential inhibitor of DNMT in several cancers.

DSF has been reported to significantly inhibit the growth and clonogenic survival of cell lines in prostate cancer by unmethylating the promoter of the APC (adenomatosis polyposis coli) gene, which encodes a tumor suppressor protein that acts as an antagonist of the Wnt signaling pathway, and the RARB (retinoic acid receptor beta) gene, which limits the growth of many cell types. DSF exposure also leads to reduction of global genomic C content (Zhao et al., 2015b). DSF in combination with other drugs can be an effective therapeutic strategy against cancer. Studies have suggested O6-methylguanine-DNA methyltransferase (MGMT) as the key factor responsible for chemoresistance of aggressive pituitary adenomas to the currently most promising chemotherapeutic drugs temozolomide (TMZ) and 2-methoxyestradiol (2ME) (Sharma et al., 2016). TMZ efficacy increases when used in combination with DSF. The antitumor effect of TMZ is observed in human pituitary cancer cells *via* the ubiquitin–proteasomal MGMT protein elimination route (Sharma et al., 2016). Estrogen receptor-β (ER-β), a tumor-suppressor gene in prostate cancer, is repressed by DNMT-mediated hypermethylation. DSF treatment reverses the silencing of ER-β and prevents cell proliferation (Wang et al., 2003). The role of DSF has been seen in the case of CRC. DSF in combination with 5-fluorouracil (5-FU) is used as the major chemotherapeutic component for CRC. It imparts chemosensitization, significantly enhanced the apoptotic effect, and synergistically potentiated the toxic effect of 5-FU on CRC cell lines. Cancer cells with high NF-кB nuclear activity demonstrate robust chemoresistance and radioresistance. DSF strongly inhibits both NF-кB nuclear translocation and DNA binding activity (Oki et al., 2007).

Inhibition of DNMT function can potentially reverse some of the cancer-associated methylation marks, reprogram the epigenetic makeup, and change the protein expression profile. DSF represents an attractive therapeutic avenue.

### Decitabine

Decitabine or 5-aza-2’-deoxycytidine (cytidine analog) is a hypomethylating agent that functions as nucleic acid inhibitors by inhibiting DNMTs. Its trade name is Dacogen. It engages the DNMTs by binding to it irreversibly through a covalent bond and inhibiting the methylation of a daughter strand during the replication (Jabbour et al., 2008). Decitabine treatment at high concentrations inhibits DNA synthesis and leads to cell cycle arrest (Palii et al., 2008), whereas its low-dose but long-term treatment eventually causes degradation of DNMTs without cell cycle arrest by getting DNMTs entrapped (Briot et al., 2017). Many studies have been carried out to increase the efficiency of decitabine as it has less oral bioavailability. To increase its oral bioavailability, lipid nanocapsules have been developed to encapsulate the decitabine. Decitabine cytotoxicity was observed to be higher when used in conjugation with lipid nanocapsules against different cancer cell lines (Briot et al., 2017).

Understanding of molecular mechanism is necessary to enhance the efficacy of any drug. Decitabine is involved in the regulation of key factors to prevent cancer. Overall, it helps in the restoration of cancer cell sensitivity toward drugs and body immunity (cytotoxic lymphocytes) and most importantly reverses the EMT. Multidrug resistance continues to be a big hurdle for cancer treatment. Decitabine restores drug sensitivity by p-glycoprotein (P-gp) coded by the mdr-1 gene in both myeloid and solid tumor cells K562/ADR and MCF-7/ADR, respectively, in a time- and dose-dependent manner (Wang et al., 2017a). It has been demonstrated that decitabine inhibits the MAPK pathway, which could be the possible reason behind upregulation of p-glycoprotein (Vitale et al., 2017). To overcome drug resistance, decitabine has been combinedly used with the mTOR inhibitor everolimus for the treatment of medullary thyroid cancer (MTC). This combination showed strong antiproliferative activity through apoptosis induction. Through bioinformatics, four major molecular pathways involved in cancer progression were seen to be affected, including PI3K-Akt signaling, ECM/receptor interaction, neurotrophin pathway, and focal adhesion, which leads to the apoptosis of cancer cells through the overexpression of apoptosis regulators NGFR and Bax genes (Li et al., 2017b). Decitabine enhances the allo-NK cell-mediated killing effects on leukemia stem cell by upregulation of NKG2D ligands (Morel et al., 2017). Decitabine is also involved in miRNA regulation, and miR-375 level is upregulated after decitabine treatment, which, in turn, represses HPV16 E6 oncoprotein level (Bai et al., 2017). Progestin and adipoQ receptor family member 3 (PAQR3) expression is significantly associated with advanced TNM stage of cancer. Decitabine treatment induced the expression of PARQ3, which significantly reduced proliferation, colony formation, and invasion of ESCC cells *via* inhibition of ERK signaling (Wang et al., 2017b). Decitabine restores tumor suppressor Bridging integrator-1 (Bin1), which reduces ESCC cell malignant behaviors and reverses EMT *via* regression of MMP-2 and MMP-9 expression through the PTEN/AKT signaling pathway.

In CRC, decitabine has been used alone as well as in combination with other drugs. Low expression of NALP1 (nucleotide-binding oligomerization domain-like receptor family, pyrin domain-containing 1) is associated with survival and tumor metastasis in colon cancer. DAC treatment increases its expression and hence suppressed the growth of colon cancer (Chen et al., 2015). Decitabine treatment suppressed the invasion ability of CRC lymph node metastasis-derived SW620 cells as well as oxaliplatin-resistant SW620 cells. Cells regained the epithelial characteristic that was indicated by upregulation of E-cadherin, miR-200C, and miR-141 (Tanaka et al., 2015; Manfrão-Netto et al., 2018). It has also been used in combination with other drugs like gefitinib and azacitidine as the effective treatment approach for CRC (Müller and Florek, 2010; Gerecke et al., 2018).

### Azacitidine

Azacitidine (marketed as Vidaza) is basically a ribonucleoside and functions as a chemical analog of cytidine. It acts as a hypomethylating agent. It gets incorporated into the RNA larger than into a DNA. Decitabine is the deoxy derivative of azacitidine. It differs from decitabine in the way that it can bind to both DNA and RNA, whereas decitabine can bind only to the DNA. Its oral version is called CC-486. It works in a dose-dependent manner. At low dose, it causes hypomethylation of DNA by inhibiting DNMT by covalent binding with it, whereas at high dose, it functions as a cytotoxic agent and gets incorporated into DNA and RNA in the abnormal cells, resulting in cell death (Borodovsky et al., 2013).

It has been demonstrated that somatic mutations in isocitrate dehydrogenase 1 (IDH1) alone sufficiently induce a global hypermethylated phenotype, which is one of the features of the glioma with this kind of mutation. Long-term treatment with azacytidine resulted in reduction of DNA methylation, which results in glial differentiation, reduction in cell proliferation, and tumor growth. Also, there was no sign of recurrence despite discontinuation of therapy (Lee et al., 2018). H3K9me3 and H3K27me3 marks are associated with cancer progression and metastasis. Azacitidine treatment resulted in reduction of H3K9me3 and H3K27me3 marks in neuroendocrine prostate cancer (Roulois et al., 2015). At low dose, azacytidine targets CRC initiating cells by induction of viral mimicry *via* the MDA5/MAVS/IRF7 pathway (Hu et al., 2017). The reduced expression of NDN (Necdin, MAGE Family Member) is associated with poor differentiation, advanced TNM stage, and poor prognosis of CRC. Administration of azacytidine causes hypomethylation of the NDN promoter. Enhancement in the expression of NDN causes it to bind to the LRP6 promoter, leading to reduced transcription and Wnt signaling pathway inhibition in CRC (Lai et al., 2015).

### Chaetocin

Chaetocin is a fungal mycotoxin that inhibits HMTs. HMT SUV39H1-mediated methylation of lysine 9 on histone H3 is associated with repression of tumor suppressor genes. Treatment of cancer cell lines with chaetocin led to downregulation of SUV39H1 along with reduction in H3K9 status (Cherblanc et al., 2013; Chiba et al., 2015). Previously, chaetocin was concluded as a “specific” inhibitor of the H3K9 HKMT (histone lysine methyltransferase) SU(VAR)3–9, but later on, it was proved that it is a non-specific inhibitor of HMTs (Zuma et al., 2017). It promotes irreversible arrest of cell cycle, nucleolus fragmentation, and RNA transcript blockage in pathogenic trypanosomatids (Dixit et al., 2014). Chaetocin induces ROS-mediated apoptosis through an ATM-YAP1-driven apoptotic pathway in glioma cells (Shuai et al., 2018). Chaetocin also shows synergistic cytotoxity in combination with other epigenetic drugs such as SAHA (HDACi) or JQ (bromodomain inhibitor) (Chiba et al., 2015).

The histone H3 lysine 9 (H3K9) methylation mark is linked with the progression of CRC and positively correlated with the metastasis (Yokoyama et al., 2013). Methyltransferases SUV39H1/SUV39H2 have been seen to be involved in cell migration regulation (Yokoyama et al., 2013; Liu et al., 2015). Chaetocin treatment was observed to reduce the cell migration in CRC cell lines (Liu et al., 2015). Thus, chaetocin alone or in combination with other drugs may be a potent drug for the treatment of multiple cancers. Chaetocin has been demonstrated to suppress cancer cells through the induction of apoptosis. In multiple lung cancer cells, its treatment activates endoplasmic reticulum stress, which results in the upregulation of ER stress response proteins, transcription factor ATF3, and CHOP, which further contributes to apoptosis in a death receptor 5 (DR5)-dependent manner (Zhao et al., 2015a). Treatment of chaetocin reduces cell growth by downregulating Blimp1 and RANKL (receptor activator of NFκB ligand) expression, which reduces osteoclast differentiation. Osteoclast gets differentiated from hematopoetic macrophage-like cells through the RANKL–RANK signaling system. Osteoclast formation is associated with the bone-responsive diseases (Zhao et al., 2015a). In B16F10 mouse melanoma cells, chaetocin inhibits IBMX (3-isobutyl-1-methylxanthine)-induced melanogenesis through activation of ERK (Bae et al., 2016). Chaetocin is able to induce autophagy along with caspase-dependent apoptosis in hepatic cancer, but inhibition of autophagy enhances its effectivity as an anticancer apoptotic agent (Jung et al., 2016). There is still a lot more to be discovered as the molecular mechanism underlying the behavior as anticancer agent in several types of cancer remains unclear.

**Figure 3:**
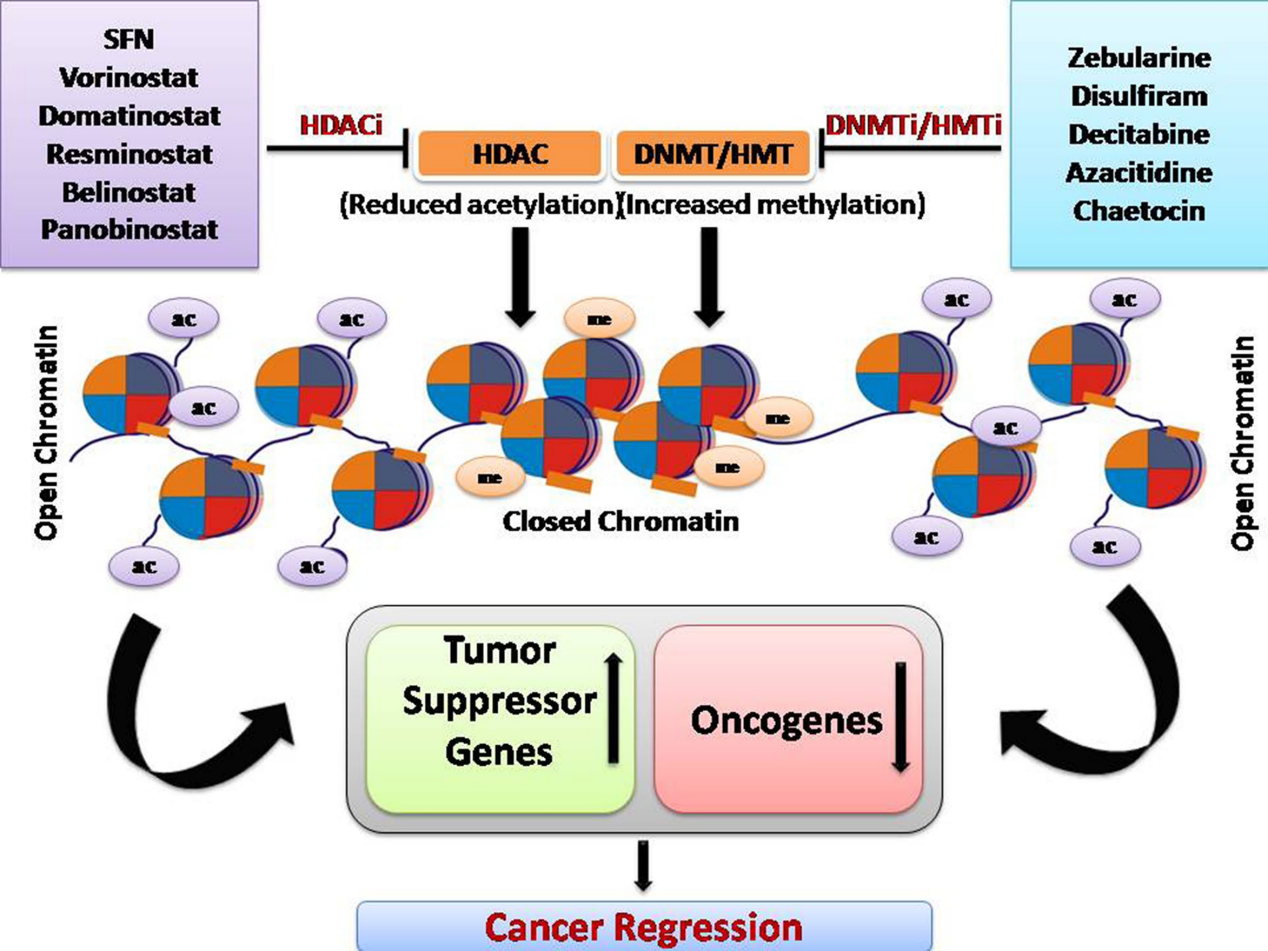
Overall mechanism of function of DNA methyltransferase inhibitors and HDAC inhibitors.

Epigenetic modification is a crucial mechanism in cancer and has been exploited for the development of anticancer therapeutic drugs. Overall, HDAC inhibitor and DNMT/HMT inhibitor treatment increases the expression of tumor suppressor genes, genes responsible for drug sensitivity, and reduced the expression of oncogenes (**Figure 3**).

## Conclusion and Future Perspective

Currently, there are several potential drugs targeting HDACs and DNA/histone methyltransferases (DNMT/HMT) used in treating several types of cancer (**Table 1**). Most of the drugs mentioned in this review are FDA-approved as they work efficiently in specific cancers at certain stages of the disease.

**Table 1:** Chemotherapeutic drugs targeting epigenetic modifications and signaling pathways.

Target epigenetic modification	Name of the drug	Cancer models	Signaling pathways affected
Histone Deacetylases Inhibitors	Sulforaphane	Leukemia, colorectal cancer, prostate cancer,	p21 pathway,
	Vorinostat	Leukemia, T-cell lymphoma, prostate cancer, bladder cancer, breast cancer	ROS-dependent apoptosis , p21 pathway
	Domatinostat	Leukemia, colorectal cancer	Hedgehog(HH)/Gli signaling pathway, ASK-1 dependent mitochondrial apoptosis pathway, TGF-β pathway
	Resminostat	Leukemia, colorectal cancer, head and neck carcinoma, hepatocellular carcinoma	AKT signaling,
	Belinostat	Leukemia, colorectal cancer, lung cancer, pancreatic cancer	MAPK signaling, TGF-β pathway,
	Panobinostat	Leukemia, colorectal cancer, head and neck cancer, Hodgkin’s lymphoma, prostate cancer, breast cancer, thyroid cancer	EGFR/HER2 signaling, MAPK signaling, PI3K-Akt, NFκB pathway.
DNA Methyltransferases Inhibitiors	Zebularine	Leukemia, colorectal cancer, lung cancer, cholangiocarcinoma, gastric cancer, pancreatic cancer, head and neck cancer, hepatocellular carcinoma, cervical cancer, breast cancer, bladder carcinoma	Caspase-dependent apoptosis, Wnt signaling pathway
	Disulfiram	Leukemia, colorectal cancer, prostate cancer, pituitary adenomas	Wnt signaling pathway, NFκB pathway.
	Decitabine	Leukemia, colorectal cancer, breast cancer, thyroid cancer, lung cancer	MAPK, PI3K-Akt, TGF-β, ERK-signaling, NGFR
	Azacitidine	Leukemia, colorectal cancer, glioma, prostate cancer	Wnt signaling, MDA5/MAVS/IRF7 pathway
Histone Methyltansferases Inhibitors	Chaetocin	Leukemia, colorectal cancer, lung cancer	NFκB, ERK-signaling, Caspase-dependent apoptosis.

Although the approach of using epigenetic modifying agents as anticancer drugs may have clinical benefits, there are several problems that must be considered. Even after the drug treatment, the reversible nature of methylation persists. The remethylation and suppleness are major problems that needed to be resolved. Moreover, it is important to consider that involvement of HDACs in cancer does not necessarily mean its overexpression. Sometimes, truncated or mutated HDACs can also be present. In such cases, alternative therapeutic drugs will be required. One of the major limitations associated with HDACs and DNMT enzymes is lack of specificity. Through the use of DNMT inhibitor therapy, non-specific genes are reactivated. The epigenome is tremendously complex; hence, it is needed to minimize the off-target effects of epigenetic modifying drugs. There is room for development of effective methods for drug delivery to reduce side effects and attain a higher therapeutic index. There are various delivery systems like nanocarriers, administering drugs in combination for synergistic effect and or altering the chemical structure of drugs, which enhance the effectiveness of the drugs. There are various nanocarriers that are used to deliver the drug, including nanogels, liposomes, dendrimers, and polymeric nanoparticles. Epigenetic drugs have less stability and hence less sustainability in the body. For example, some drugs like decitabine need to be administered continuously as it is rapidly degraded in the body and system drug level drops. Drug delivery modification enhances drug stability, permeability and retention. It also lowers the required drug concentration during administration. During DNA replication, inhibition of DNMT distorts methylation status throughout the genome. Methylation targets many DNA repair pathway genes, whose expression may lead to drug resistance. During chemotherapy, cancer stem cells are a small population of cells that escape and enter into the dormancy phase, slow the growth rate, and resemble normal stem cell properties. These residual cancer stem cells, after getting triggered, appear as a recurrent and chemoresistance disease. Combined therapies of standard chemotherapeutic drugs with epigenetic targeting drugs provide the opportunity for the reactivation of genes required for response to the chemotherapeutic drugs. Combining epigenetic therapies has shown both additive and synergistic effects. One of the main side effects of these drugs is their toxic effect. Many epigenetic modification targeting drugs like demethylating agents azacytidine and decitabine are highly toxic in nature. However, it is not easy to develop a chemically derived drug that is not toxic to normal cells. Therefore, drug discovery using plant species and their natural products is drawing attention. Being naturally derived drugs, they have been shown to have less or non-toxic effects on normal cells and are more tolerable. Their anticancer properties and natural abundance make them a great candidate for drug development. Plant-derived drugs can also be categorized into four classes according to their activities: antioxidants, cell cycle inhibitors, methyltransferase inhibitors, and HDAC inhibitors. For example, SFN, isoflavones, and isothiocyanates work as HDACi. There are many plant-derived natural compounds and secondary metabolites still under investigation for their anticancer activities and can be the scope for development of new clinical drugs.

## Author Contributions

SP conceptualized the topic, framed the write-up sequence, collected the relevant references, and finalized the manuscript. A contributed in writing some of the portions under the guidance of SP.

## Conflict of Interest Statement

The handling editor declared a past co-authorship with one of the authors. The authors declare that the research was conducted in the absence of any commercial or financial relationships that could be construed as a potential conflict of interest.
